# Yellowstone Lake *Nanoarchaeota*

**DOI:** 10.3389/fmicb.2013.00274

**Published:** 2013-09-11

**Authors:** Scott Clingenpeel, Jinjun Kan, Richard E. Macur, Tanja Woyke, Dave Lovalvo, John Varley, William P. Inskeep, Kenneth Nealson, Timothy R. McDermott

**Affiliations:** ^1^DOE Joint Genome InstituteWalnut Creek, CA, USA; ^2^Stroud Water Research CenterAvondale, PA, USA; ^3^Center for Biofilm Engineering, Montana State UniversityBozeman, MT, USA; ^4^Eastern OceanicsWest Redding, CT, USA; ^5^Montana Institute on Ecosystems, Montana State UniversityBozeman, MT, USA; ^6^Department of Land Resources and Environmental Sciences, Montana State UniversityBozeman, MT, USA; ^7^Thermal Biology Institute, Montana State UniversityBozeman, MT, USA; ^8^Department of Earth Sciences, University of Southern CaliforniaLos Angeles, CA, USA; ^9^J. Craig Venter InstituteSan Diego, CA, USA

**Keywords:** *Nanoarchaeota*, Yellowstone Lake, pyrosequencing

## Abstract

Considerable *Nanoarchaeota* novelty and diversity were encountered in Yellowstone Lake, Yellowstone National Park (YNP), where sampling targeted lake floor hydrothermal vent fluids, streamers and sediments associated with these vents, and in planktonic photic zones in three different regions of the lake. Significant homonucleotide repeats (HR) were observed in pyrosequence reads and in near full-length Sanger sequences, averaging 112 HR per 1349 bp clone and could confound diversity estimates derived from pyrosequencing, resulting in false nucleotide insertions or deletions (indels). However, Sanger sequencing of two different sets of PCR clones (110 bp, 1349 bp) demonstrated that at least some of these indels are real. The majority of the *Nanoarchaeota* PCR amplicons were vent associated; however, curiously, one relatively small *Nanoarchaeota* OTU (71 pyrosequencing reads) was only found in photic zone water samples obtained from a region of the lake furthest removed from the hydrothermal regions of the lake. Extensive pyrosequencing failed to demonstrate the presence of an *Ignicoccus* lineage in this lake, suggesting the *Nanoarchaeota* in this environment are associated with novel *Archaea* hosts. Defined phylogroups based on near full-length PCR clones document the significant *Nanoarchaeota* 16S rRNA gene diversity in this lake and firmly establish a terrestrial clade distinct from the marine *Nanoarcheota* as well as from other geographical locations.

## Introduction

Huber et al. (2002) described the cultivation of a novel hyperthermophilic archeaon coined *Nanoarchaeum equitans*. This organism requires the host organism *Ignicoccus hospitalis*, living as an obligate parasite because it lacks genes coding for biosynthesis of essential cellular components such as lipids, cofactors, amino acids, or nucleotides (Waters et al., 2003). Thus far, *N. equitans* is the lone cultured representative of the archaeal subdivision *Nanoarchaeota*, though PCR-based environmental studies have found the *Nanoarchaeota* in several high temperature marine environments (Hohn et al., 2002; Stetter et al., 2005; McCliment et al., 2006; Roussel et al., 2011; Flores et al., 2011, 2012). In addition, the *Nanoarchaeota* 16S signature has been documented in hypersaline mats (Casanueva et al., 2008), suggesting these organisms are more widely distributed than in geo/hydrothermal environments.

Yellowstone National Park (YNP) is a well-known high temperature environment that has been extensively studied, in particular during the last decade. In addition to being home to a wide range of organisms belonging to the domain *Bacteria* (e.g., Hugenholtz et al., 1998; Fouke et al., 2000; Reysenbach et al., 2000; Jackson et al., 2001; Botero et al., 2005; Spear et al., 2005; Yang et al., 2011), documentation of the *Archaea* in the YNP geothermal microbial communities has also been accumulating (e.g., Barns et al., 1994; Karavaĭko et al., 1994; Auchtung et al., 2006; Boyd et al., 2007; Ellis et al., 2008; Inskeep et al., 2010; Kan et al., 2011). Thus, far, three reports of the *Nanoarchaeota* have been noted for YNP, with all involving work at Obsidian Pool. Hohn et al. (2002) PCR cloned *Nanoarchaeota* 16S rRNA genes from Obsidian Pool, referred to as clone OP9. Later, Stetter et al. (2005) examined Obsidian Pool samples with *Nanoarchaeota*-specific FISH probes and described “tiny cocci, about the size of *N. equitans* attached to the surface of Pyrobaculum-shaped rods that may represent these novel nanoarchaeotes.” And just recently, Podar et al. (2013) used cell sorting techniques to capture and genome sequence *Nanoarchaeota* cells (coined Nst1) and their inferred hosts from Obsidian Pool, YNP. This latter study represents an important advancement in *Nanoarchaeota* biology in that the genome comparison with that of *N. equitans* revealed clear differences (e.g., less reduction). Importantly, Podar et al. (2013) also provided evidence of the *Nanoarchaeota* associated with host *Archaea* different from *Ignicoccus*, inferred to be a *Sulfolobales*-like organism in this case (Podar et al., 2013).

While these contemporary efforts have made foundational changes to our understanding of the microbial diversity and distribution in the YNP geothermal complex, many geotherm features remain to be studied. One such environment is Yellowstone Lake. It is the highest elevation (~2300 m) among large subalpine high-altitude lakes in North America (Morgan et al., 2007), with a maximum measured depth of 131 m and average depth of 42.5 m (Benson, 1961; Morgan et al., 2007). Several studies conducted by the United States Geologic Survey (USGS) have documented extinct or active hydrothermal vents at specific locations on the lake floor (Morgan et al., 2003; Balistrieri et al., 2007; Morgan et al., 2007). Thus, in addition to the microbiota that normally comprise the microbial community of a sub-alpine lake, there are substantial opportunities to study thermophiles associated with the lake floor vents.

We have recently conducted extensive surveys of this lake (Clingenpeel et al., 2011; Kan et al., 2011), characterizing its high-energy geochemistry and substantial microbial diversity, and documenting microbial phylotypes previously known to only occur in marine environments. Here we describe additional work with the vents and photic zones in this lake, though in this case targeting the *Nanoarchaeota*. We describe the geochemistry and summarize the results from pyrosequencing and Sanger sequencing of 16S rRNA gene clones generated with *Nanoarchaeota* specific PCR primers. The cloned sequences document a North American clade of the *Nanoarchaeota* and the significant diversity therein.

## Materials and methods

### Lake locations and sampling

**Figure 1:**
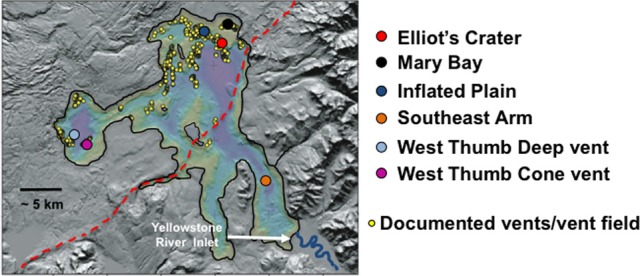
**A relief map illustrating Yellowstone Lake and the approximate sampling locations described in this study.** Image is a modification from that published in Morgan et al. (2007) and is used here with permission. Red dashed line represents the approximate boundary of the Yellowstone caldera. Relief map image kindly provided by Lisa Morgan.

Lake sampling took place in September 2007 and 2008. Vent fields were located based on global information system coordinates established from past USGS surveys (e.g., Morgan et al., 1977, 2007). Individual vents were located and sampled by remote operated vehicle (ROV) reconnaissance of the lake floor within the Inflated Plain and West Thumb regions of the lake. Lake location and brief description of each site and of within-site samples are described below, and the relative and approximate lake locations are shown in Figure 1. Vent fluids and streamer samples were collected using a boat-tethered ROV previously described (Lovalvo et al., 2010; Clingenpeel et al., 2011; Kan et al., 2011). Characterization for aqueous solutes and gases were as recently described (Clingenpeel et al., 2011).

Biomass sampling methods were as described by Clingenpeel et al. (2011). Briefly, 100–300 l of lake or vent water was pumped through a 20 μm pre-filter into 50 l carboys on the boat deck. Carboys were sterilized prior to use by autoclaving or by bleaching followed by rinsing with autoclaved distilled water. Using techniques previously described for the global ocean survey (Rusch et al., 2007), the biomass in the lake and vent water was size fractionated by serial filtration through 3.0, 0.8, and 0.1 μm membrane filters. Filters were sealed in plastic bags and frozen for transport to the laboratory at Montana State University, where they were stored at −80°C. To obtain microbial streamer samples from the vent flow stream, the ROV sampling arm was positioned over the streamer structure and then an ROV vacuum device was engaged to suction the streamer biomass into a holding canister on the ROV (see Lovalvo et al., 2010).

### Nucleic acid extraction, PCR, and sequencing

DNA was extracted as described by Clingenpeel et al. (2011). Full-length 16S rRNA gene amplification for clone library construction and generation of shorter amplicons for pyrosequencing were performed using primers described in Table 1. Near full-length PCR products were cloned using the TOPO TA Cloning Kit (Invitrogen Corp.), with inserts sequenced using the Big Dye Terminator chemistry (Applied Biosystems) and the Applied Biosystems 3100 Genetic Analyzer. Chimeric sequences were screened by the “CHIMERA DETECTION” program of the Ribosomal Database Project Maidak et al. (1997) and removed from further analysis.

**Table 1:** **Primers used in this study**.

**Primer**	**Sequence (5′–3′)**	**Target groups**	**References**
**FULL-LENGTH CLONING**			
N3F	TCCCGTTGATCCTGCG	Nanoarchaeota	Huber et al., 2002
N1406R	ACGGGCGGTGAGTGCAA	Nanoarchaeota	Huber et al., 2002
YNP 35F	TCCCTCCGACTAACCCATGG	YNP Nanoarchaeota	This study
YNP 1337R	ACCGGGGGAATAGTGACC	YNP Nanoarchaeota	This study
**PRIMERS FOR PYRO SEQUENCING**			
N3aF^*^	TCCCGTTGATCCTGCGG	Nanoarchaeota	This study
N3bF^*^	TCCAGTTGATCCTGCGGG	Nanoarchaeota	This study
N3cF^*^	TCCCGTGTGATCCTGCG	Nanoarchaeota	This study
N495R	TGGCGACTGCCACCCCT	Nanoarchaeota	This study

* Modified from N3F.

The V1 + V2 region of the 16S rRNA gene was amplified for 454 Titanium pyrosequencing using primers developed in this study (Table 1). After 25 cycles of amplification, 5 more cycles were used to add the sample specific barcodes and the adaptor sequences required for 454 pyrosequencing. The barcoded 16S rRNA gene PCR amplicons obtained from the different environments were pooled, with the volume of each sample qualitatively adjusted to reflect the strength of the amplicon.

Near full-length clone libraries were aligned, trimmed, and then initially classified using BLAST (Altschul et al., 1990), and can be found as GenBank accession numbers JF262403–JF262535. Neighbor-Joining distance trees were constructed using MacVector 10.0 software package (GCG) and Maximum Likelihood trees were constructed using PhyML web interface (http://www.atgc-montpellier.fr/phyml/). In both analyses, bootstraps were generated from 1000 resampling datasets. OTU groupings were assigned using ARB software (Ludwig et al., 2004) and the latest released Silva 102 database (Pruesse et al., 2007). The pyrosequencing reads were quality trimmed according to Kunin et al. (2010) followed by clustering using abundance-sorted preclustering per Huse et al. (2010) and a final complete linkage (furthest neighbor) clustering using the mothur software (Schloss et al., 2009). Collector's curve analysis was also done in mothur. The identification of pyrosequencing reads as nanoarchaea was done by classification with the RDP Classifier (Wang et al., 2007; Cole et al., 2009). Techniques we previously described (Clingenpeel et al., 2011; Kan et al., 2011) were used to match the pyrosequencing reads with the near full-length Sanger sequenced clones. Briefly, the pyroreads were compared to the near full-length clone sequences using BLAST (Altschul et al., 1990), with match criteria requiring ≥99% identity for ≥95% of the read length in order to be assigned to a phylogroup. The pyroreads can be found under the identifiers SRS150246 and SRS150227 in the SRA database.

## Results

### Sampling sites and geochemistry

The lake sampling sites examined in this study are shown in Figure 1. Two sampling sites were located in the West Thumb region and three sites in the northern portion of the lake, referred to as Elliot's Crater (a lake floor geologic feature), Mary Bay, and Inflated Plain. All of these locations correspond to lake floor vent fields previously documented by (Morgan et al., 2003, 2007) (Figure 1) and on which we have reported on recently (Lovalvo et al., 2010; Clingenpeel et al., 2011; Kan et al., 2011). As a control environment to contrast with the vent field regions of the lake, one site in the Southeast Arm was also included. This location is well-outside the caldera boundary, not known to be associated with any vent activity (Morgan et al., 2003, 2007), and most proximal to the primary tributary to the lake, the Yellowstone River. Prior to flowing into the Southeast Arm, the Yellowstone River does not drain geothermal features elsewhere in YNP (YNP Ground Surveys, Spatial Analysis Center 2005; Savage et al., 2012).

Geochemical parameters of significance to microbial selection are summarized in Table 2. Vent emissions varied in pH (5.2–6.7) and temperature (37–73°C), with the latter also exhibiting within-vent variation documented as temperature surges determined by real time ROV monitoring during sample acquisition. Gas composition varied between and within vent fields (Table 2). As examples, vent H_2_ levels in the northern regions of the lake (Inflated Plain, Mary Bay, Elliot's Crater) were consistently much greater than in the West Thumb vents (Table 2). The photic zone water chemistry varied, depending on whether the samples were acquired in water columns overlying lake floor vents and in such cases reflected the constituents observed in the vent emissions located directly below. For instance, in 10 m photic zone samples associated with the high output Inflated Plain vents (Table 2), levels of CH_4_, H_2_, and CO_2_ were orders of magnitude higher and pH more acidic (6.1–6.6) than the Southeast Arm photic waters (Table 2), which were neutral pH, cold and well-aerated. It is worth noting that NH_4_, CO_2_, CH_4_, and H_2_ in the Southeast Arm were still at microbially relevant concentrations.

**Table 2:** **Sample identification, lake location, and general characteristics. Some geochemical parameters were determined in duplicate**.

**Lake ID no.**	**Pyrosequencing reads**	**Lake location and sample type**	**Temp.(C°)**	**pH**	**Depth (m)**	**Selected nutrients and energy sources**					
						**NH_4_**	**CO_2(aq)_**	**S^2−^_(aq)_**	**O_2(aq)_**	**CH_4(aq)_**	**H_2(aq)_**
						***uM***	***mM***	***uM***	***uM***	***uM***	***uM***
**ELLIOT'S CRATER**											
1	1672	Elliot's Crater Vent emissions	63–68	6.4	14.1	45.0	0.49,47	21.7	119	2.5, 2.1	762, 558
**MARY BAY**											
2	2186	Vent steamers and sediments	62–82	5.2	50.5	53.8	3.77, 1.80	79.5	bd	28.1, 12.4	2984, 2797
**INFLATED PLAIN**											
3	257	IP photic 10 m 0.1 um filter	12.2	6.1	10	2.6	0.11, 0.11, 0.10	2.5	261	2.4, 2.7, 2.6	773, 798, 716
4	1553	IP photic 10 m 0.8 um filter									
5	252	IP photic 10 m 3.0 um filter									
6	3221	IP vent steamer 1	40–60	5.2	30	30.9	3.1, 3.2	248	bd^++^	20.9, 22.5	1031, 1430
7	2510	IP steamer 2									
8	2222	IP vent 2 emissions	44–52	5.6	33.6	8.2	1.1, 1.1	98	bd	6.7, 5.4	1974
9	1996	IP mixing zone water	21–30	5.6	33.3	37.5	0.57	118	23	2.7	386
**WEST THUMB**											
10	3309	WT deep vent emissions (2007)	60–66	6.2	52.0	12.1	1.7	2.1	113	6.4	49
11	2797	WT deep vent emissions (2008)	40–73	6.0	52.3	23.4	2.4	11.0	197	7.6	32
12	1959	WT deep vent steamer	40–73	6.0	52.3	23.4	2.4	11.0	197	7.6	32
13	1904	WT deep vent mixing zone water	26	6.6	52.1	27.2	ND^*^	0.3	211	ND	ND
14	638	West thumb cone vent	37	6.7	26.1	27.4	10.22	bd	82	7.6	14
**SOUTHEAST ARM**											
15	377	SEA photic 10 m 0.1 um filter	12.3	7.0	10.0	6.2	0.02	0.1	273	0.1	33
16	1221	SEA photic 10 m 0.8 um filter									
17	412	SEA photic 10 m 3.0 um filter									

* ND, Not determined.

++ bd, Below detection.

### Nanoarchaeote diversity: near full-length sanger sequencing

**Figure 2:**
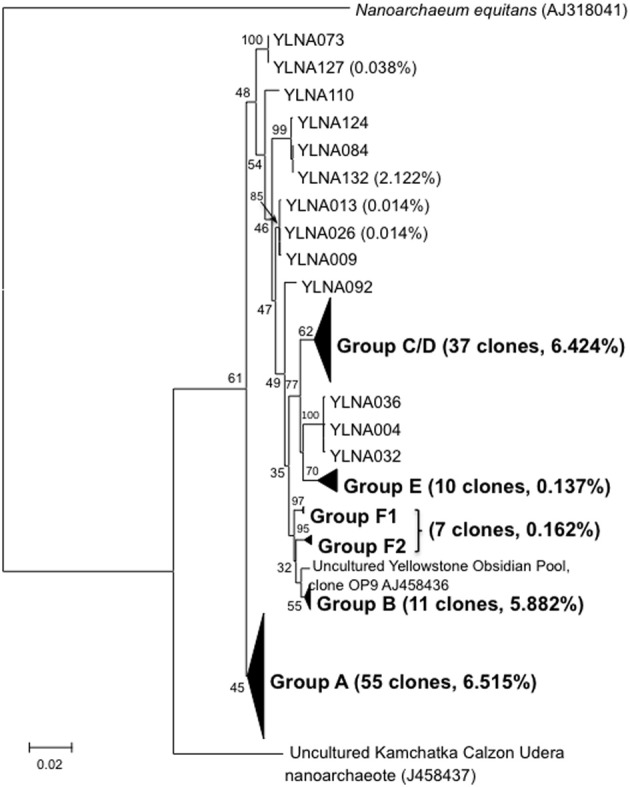
**Maximum likelihood tree illustrating the phylogenetic relatedness of the Yellowstone like near full-length *Nanoarchaeota* clones relative to other near full-length clones and to *N. queans*.** For each clone group, the number of near full-length clones obtained in this study and the percentage of proofreads that match the Sanger Sequence is provided in parentheses. Only relevant bootstrap values are shown.

A total of 131 near full-length *Nanoarchaeota* 16S rDNA PCR clones were obtained from vent or photic zone water samples. Sanger sequencing revealed considerable within-lake diversity (Figure 2). All lake clones were most closely related to the YNP *Nanoarchaeota* clones derived from Obsidian Pool (Hohn et al., 2002) located ~8 km from the lake. Further, all YNP clones branched distinctly separate from *N. equitans* and from the Kamchatka environmental clones (Figure 2). These near full-length clones were grouped based on bootstrap-supported branching cluster relatedness and designated as clone groups A–F (Figure 2). When examined using the neighbor-joining algorithm, phylogroups C and D are separate clades and phylogroup F is a single group. However, when examined using maximum likelihood, phylogroups C and D merge and phylogroup F splits into two smaller clusters (Figure 2). Consensus sequences were generated for each phylogroup (nucleotide assignments based on majority rule) and then compared and used to generate a lake-wide consensus sequence. Comparing the phylogroup consensus sequences against the lake-wide consensus sequence illustrated a total of 136 points of sequence divergence scattered across the cloned region, but with ~55% of the diversity occurring in the 550–850 nt region of the near full length clones. There were many instances of insertions and deletions (indels) observed in these comparisons.

### Nanoarchaeote diversity: pyrosequencing

The PCR amplicons from the different samples varied in strength, with the strongest amplicons deriving from samples associated with vents (fluid emissions, streamers, or mixing zones where vent fluids mixed with lake water). After quality trimming, 28,441 pyrosequencing reads were advanced to further analysis. Read number for each sample (Table 2) reflects the amplicon strength for each sample that then determined how much of each were pooled for the barcoded pyrosequencing effort. The resulting sequence microdiversity was significant, again with a high frequency of apparent indels. To further investigate these indels, additional PCRs were conducted to individually clone and Sanger sequence a 113 bp region of the *Nanoarchaeota* 16S gene (positions 365–478 in the N. equitans 16S gene, primarily the conserved region between V2 and V3). From a small sample (31 clones), many of the indels found in the pyrosequencing reads could be identically matched with the Sanger sequenced longer clones (results not shown). Since homonucleotide repeats (HRs) may contribute to these indels in pyrosequenced DNA, the near full-length Sanger-sequenced clones were examined in more detail for this feature. There were 112 ± 4 HRs of 3–7 bp in length occurring across the near full-length clones. Further analysis of these near full-length clones identified 79 indels associated with HRs that might otherwise be interpreted as potential errors if encountered in a pyrosequencing library.

**Figure 3:**
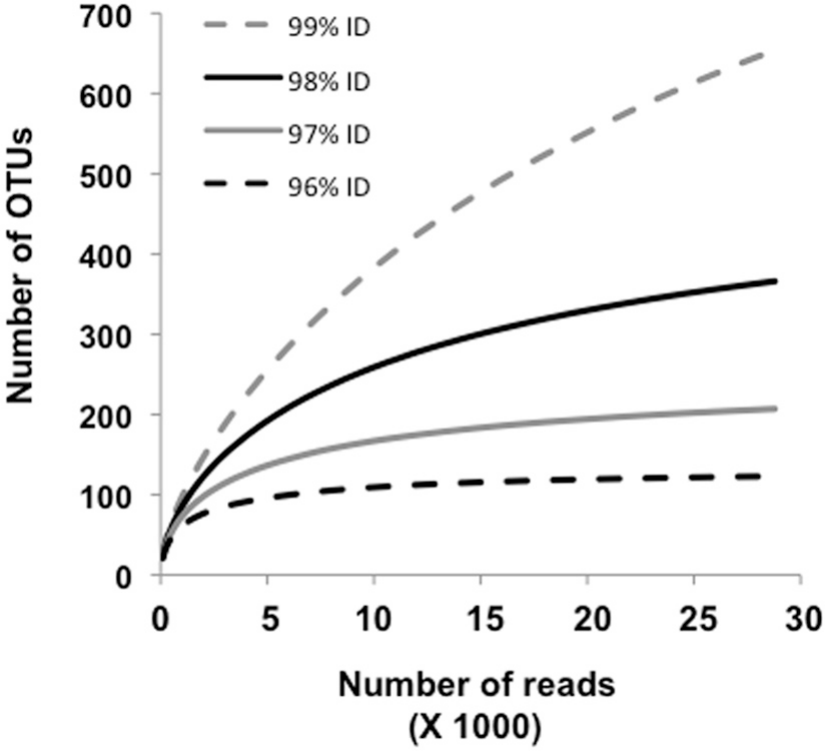
**Collector's curves estimating the number of Nanoarchaeota OUTs identified for all samples, and as a function of sequence identity set at 96, 97, 98, and 99%.** The curves depict the complete pyrosequencing data set after quality trimming according to Kunin et al. (2010) and OUT clustering as described by Huse et al. (2010).

Approximately 19% of the pyrosequences matched the near full-length PCR clones that comprised the different phylogroups (Figure 2). Of these, ~83–100% (depending on phylogroup) were associated with vent emissions, streamers or mixing zone samples (i.e., high temperature samples). For the balance of the pyrosequence reads (~81%), collector's curves were constructed to identify a conservative OTU clustering criterion. As expected, as OTU clustering criteria became more conservative total diversity estimates declined (Figure 3), with the collector's curve constructed for 96% identity suggesting that the pyrosequencing data captured a majority of the *Nanoarchaeaota* diversity in the lake sampling scheme that spanned north-south and east-west, as well as various hydrothermal features. Taking into account all habitat types (photic zone, vents, vent-associated streamers, and vent-lake water mixing zones) and examining the lake by region, most of the OTUs were found in the West Thumb and Inflated Plain regions of the lake, again corresponding to the lake floor hydrothermal vents, which are primarily found in these regions of the lake (Figure 1).

Interestingly, even at the conservative OTU clustering employed, there was single, relatively small OTU (70 reads) that was unique to the photic zone sample taken in the Southeast Arm (Figure 1), a region of the lake where no known vent activity exists. This particular OTU was also exclusive to the largest size-class biomass (smaller than 20 μm but larger than 3.0 μm) and did not group with any of the major phylogroups identified using near full-length sequences (discussed above, Figure 2). The cohesiveness of this OTU was further examined by assessing the shared identity of the reads at higher levels of sequence identity. At 99%, this OTU disaggregated to single groups of 33 reads and 12 reads, four groups of four reads each, four groups of two reads each, and one singleton. At 98% identity, it broke into three groups of 49, 16, and 5 reads, whereas at 97% identity it remained complete at 70 reads.

**Figure 4:**
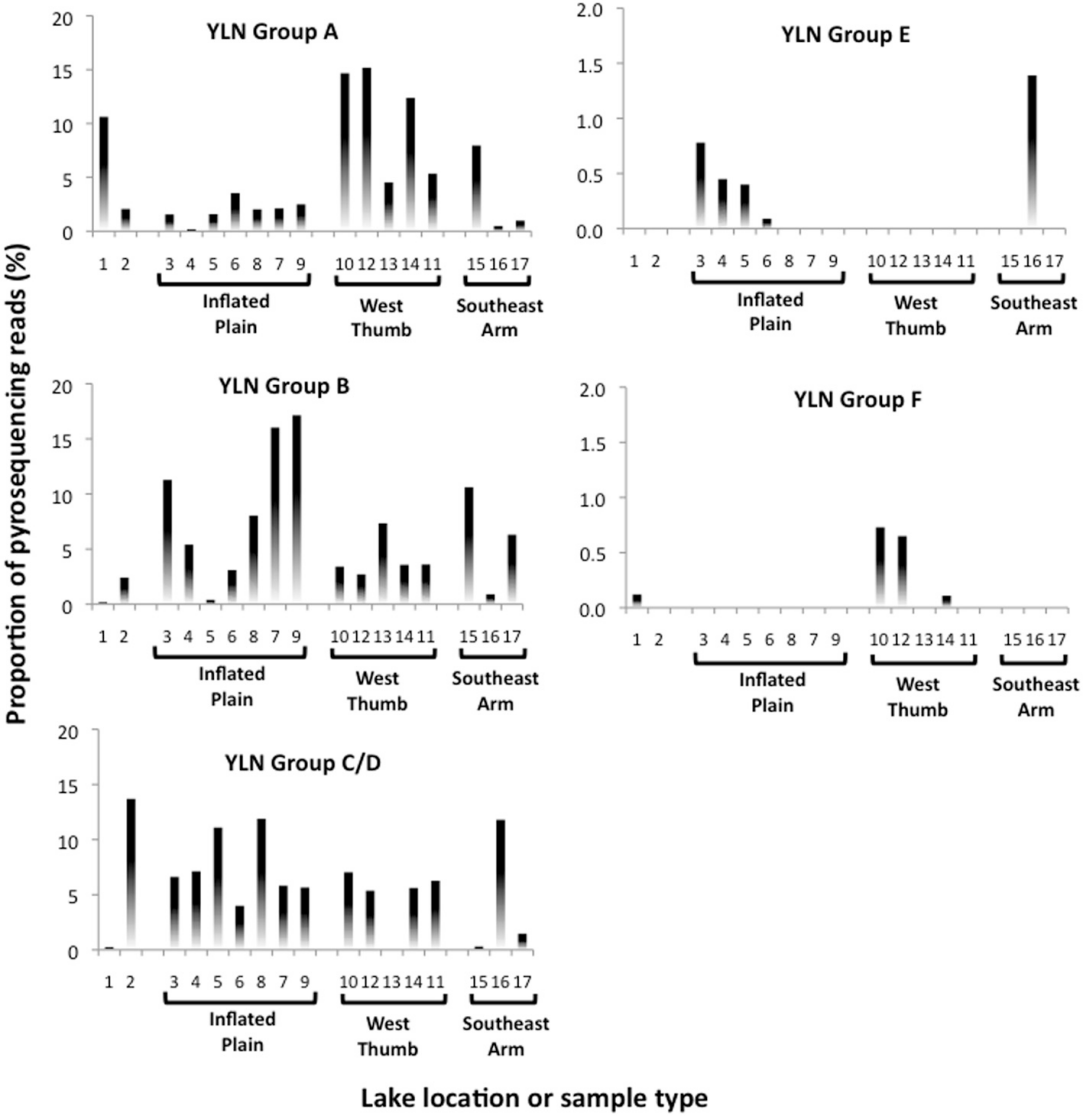
**Nanoarchaeota phylogroup distribution across Yellowstone Lake based on 99% pyroread sequence match to near full length Sanger-sequenced clones shown in Figure 2.** Data shown the proportional representation of each phlyogroup in the pyrosequence library generated for each site or sample. Location/sample numbers are keyed to Table 1, which provides a detailed description for each environment. Note the different *y*-axis scales.

Photic zone water samples from the West Thumb region failed to generate a PCR product, suggesting that at least at the time of sampling the *Nanoarchaeota* were absent or below PCR detection in this portion of the lake. Other potentially interesting distribution patterns were revealed when examining pyrosequence distribution as matched to the phylogroups identified in the near full length clones (see Figure 2). Whereas phylogroups A, B, and C/D were found throughout the lake (Figure 4), phylogroups E and F signatures appeared to exhibit patterns. For example, phylogroup E was predominantly (90% of the group E matching pyroreads) found in photic zone samples in the Inflated Plain and in particular the Southeast Arm, but was not detectable in the West Thumb vents nor in the Elliot's Crater or Mary Bay vent emissions (Figure 4). By contrast, phylogroup F (F1 and F2 combined for analysis) was primarily (95%) found associated West Thumb vents, but was undetectable in any of the Inflated Plain vent or photic samples, nor in the Southeast Arm photic water samples (Figure 4).

Finally, in an attempt to gain more information about the *Nanoarchaeota* in this lake, fluorescent *in situ* hybridization (FISH) probes were designed, prepared, and applied to raw lake photic water samples. These FISH probing attempts were hampered by visual interference from small sized lake debris particles and presumably low target density. The latter was concluded from the weak *Nanoarchaeota* PCR amplicon strength in these samples relative to the much more robust *Bacteria* or *Archaea* 16S rRNA gene amplification products observed in previous studies on this lake (Clingenpeel et al., 2011; Kan et al., 2011).

## Discussion

The *Nanoarchaeaota* are represented by a single co-cultured and characterized isolate, *N. equitans* (Huber et al., 2002). As such, it is unreasonable to assume that this lone isolate adequately represents this proposed phylum. Indeed, a recent single cell genomics study highlighted differences between *N. equitans* and *Nanoarchaeaota* cells captured from Obsidian Pool in Yellowstone (Podar et al., 2013). Initial views of the *Nanoarchaeaota* being hyperthermophiles associated with *Ignicoccus* have given way to subsequent molecular-based surveys that found the *Nanoarchaeaota* occurring in extreme environments where *Ignicoccus* is not known to reside (Casanueva et al., 2008; Podar et al., 2013). As environmental clone data has accumulated, biogeographical patterns have begun to emerge, which were considerably strengthened and expanded by the current study. The clone sequences acquired in this study firmly establishes the Yellowstone *Nanoarchaeaota* as a robust and distinct phylogenetica clade separate from those in geographically distant locations (Figure 2).

Indels were not the primary source of sequence diversity that defined the primary phylogroups observed in this lake (Figure 2), but their frequency was significant. Some indels in the pyrosequencing libraries were no doubt errors associated with HRs (Kunin et al., 2010), which are very dense in the *Nanoarchaeota* 16S gene sequences examined in this study. As averaged from six randomly selected near full-length clones, the frequency of HR per clone was: 3 bp *HR* = 73 ± 2; 4 bp *HR* = 21 ± 3; 5 bp *HR* = 11 ± 1; 6 bp *HR* ~1 ± 1; or 7 bp *HR* = 0.3 ± 0.6. As a contrast example, this is roughly two-fold that found across the longer (1542 bp) length of the seven 16S rRNA genes of *Escherichia coli* strain K12: 3 bp *HR* = 55 ± 2; 4 bp *HR* = 11 ± 2; 5 bp *HR* = 4 ± 0; 6 bp *HR* ~1 ± 0; or 7 bp *HR* = 0. Regardless of the HR issue, however, Sanger sequencing of clones from two different PCR libraries demonstrated that some of these indels appear real. This conclusion is based on multiX coverage for nucleotide assignments that appeared as an indel relative to other clone sequences and suggests the indels are a natural feature of the *Nanoarchaeota* 16S rRNA gene. For logistical and cost reasons, the absolute frequency of these indels would be very difficult to pinpoint.

Rarefaction analysis set at 96% identity suggested complete coverage of the lake *Nanoarchaeota* as defined by the pyrosequencing library (Figure 3). A small proportion (~2%) of the 96% identity defined OTUs were detected in all lake samples examined, suggesting some level of lake-wide mixing and is consistent with what was observed with pyroreads assigned to phylogroups A, B, and C/D (Figure 4) as well as what we have reported for the *Bacteria* (Clingenpeel et al., 2011) and *Archaea* (Kan et al., 2011) in this lake. And while the pyrosequences that matched all of the major phylogroups depicted in Figure 2 were primarily found associated with the lake floor hydrothermal features (Figure 4), there were instances such as with phylogroups E and F where abundance appeared to be biased toward photic zone waters (Figure 4). Further, the pyrosequencing libraries contained a single OTU that was not found in the high temperature samples, but rather only in the largest filtration size class and only in the Southeast Arm photic zone waters. *Nanoarchaeota* being associated with the lake floor hydrothermal vents was not necessarily unanticipated, but the potential for photic zone *Nanoarchaeota* was not expected. Occurrence of the *Nanoarchaeota* in low temperature environments has been documented previously (Casanueva et al., 2008), establishing a precedent for low temperature versions of this interesting microorganism. This will be the subject of follow-up work.

At this juncture, linking the Yellowstone Lake *Nanoarchaeota* to potential host phylotypes is not possible, except to conclude with near certainty that the potential host list does not include an *Ignicoccus*-like lineage. Our previous studies of this lake yielded several lines of evidence demonstrating freshwater parallels to important marine organisms; e.g., *Prochloroccus* (Clingenpeel et al., 2011) and a *Nitrosopumilus*-like archaean (Kan et al., 2011). However, an *Ignicoccus*-like lineage was notably absent in pyrosequencing surveys totaling 51,017 454-FLX reads (Kan et al., 2011) and 262,173 454-Titanium reads [Community Cyberinfrastructure for Advanced Microbial Ecology Research & Analysis (CAMERA); https://portal.camera.calit2.net/gridsphere/gridsphere?cid=microgenome]. Preliminary evidence of *Nanoarchaeota* associated (physically attached) with a *Pyrobaculum*-shaped bacterium (Stetter et al., 2005) is consistent with the view that other *Archaea* can serve as hosts. Further, a recent report by Podar et al. (2013) described a relationship between *Nanoarchaeota* from Obsidian Pool (YNP) and a *Sulfolobales*-like archaeon that co-isolated in cell sorting experiments used for single cell genome sequencing efforts. Non-*Ignecoccus* hosts would also seem the case for the *Nanoarchaeota* documented for non-thermal hypersaline mats (Casanueva et al., 2008).

In summary, this study revealed the very significant *Nanoarchaeota* 16S rRNA gene diversity occurring in natural populations associated with the hydrothermal vents in Yellowstone Lake as well as lineages that may reside in photic waters. Phylogenetically, these organisms form a clade that clusters with the YNP Obsidian Pool *Nanoarchaeota* clone that is distinctly separate from the *N. equitans* and the Kamchatka *Nanoarchaeota*. The hosts for the Yellowstone Lake *Nanoarchaeota* are unknown at present, but we conclude do not include *Ignicoccus*.

### Conflict of interest statement

The authors declare that the research was conducted in the absence of any commercial or financial relationships that could be construed as a potential conflict of interest.
